# Morphological and Immunohistochemical Examination of Lymphoproliferative Lesions Caused by Marek’s Disease Virus in Breeder Chickens

**DOI:** 10.3390/ani10081280

**Published:** 2020-07-27

**Authors:** Alessandro Stamilla, Antonino Messina, Lucia Condorelli, Francesca Licitra, Francesco Antoci, Massimiliano Lanza, Guido Ruggero Loria, Giuseppe Cascone, Roberto Puleio

**Affiliations:** 1 Dipartimento di Agricoltura, Alimentazione e Ambiente (Di3A), University of Catania, Via Valdisavoia, 5, 95123 Catania, Italy; m.lanza@unict.it; 2 DVM Consultant Poultry Specialist, via Cava Gucciardo Pirato, 12, 97015 Modica, Italy; vetmessina@gmail.com; 3 Istituto Zooprofilattico Sperimentale della Sicilia; Via Gino Marinuzzi, 3, 90129 Palermo, Italy; francescalicitra15@gmail.com (F.L.); francesco.antoci@izssicilia.it (F.A.); guidoruggero.loria@izssicilia.it (G.R.L.); giuseppe.cascone60@gmail.com (G.C.); roberto.puleio@izssicilia.it (R.P.)

**Keywords:** Marek’s disease, broiler, pathohistology, immunohistochemistry, CD3, virus

## Abstract

**Simple Summary:**

The poultry industry is the most intensive and fastest growing among all livestock production systems, and, in the last decades, it has expanded exponentially due to an increasing demand for meat and eggs. Marek’s disease is a highly contagious and rapidly progressive lymphoproliferative disease. It is one of the most dangerous diseases of those affecting the sector because it causes important economic losses. Although widely controlled by vaccination programs, sometimes chickens are not totally protected, and the presence of virulent field strains can allow outbreaks. This case describes the occurrence of Marek’s disease observed in a breeder chicken flock that reported an increase in mortality rate (+0.4–0.6%) after the 32nd week. Histological analysis has highlighted severe lesions on visceral organs of chickens caused by Marek’s disease, especially in the intestinal tract of a hen that had a tumor mass in the distal part of the cloaca. Immunohistochemical staining confirmed the disease-associated tumor. The aim of this study was to underline the importance of vaccine administration related to the maintenance of proper biosecurity practice, especially in the first week of the raising cycle. In addition, monitoring for disease even after vaccination is crucial to minimize economic loss.

**Abstract:**

Marek’s disease is widely controlled by vaccination programs; however, chickens are not totally protected, especially immediately after the vaccination when a strong challenge could interfere with the effectiveness of vaccination in the absence of proper biosecurity practice. This case report describes the occurrence of Marek’s disease (MD) observed in a breeder chicken flock reared southeast of Sicily. MD outbreak occurred from 32 to 47 weeks with an increase in weekly mortality rate (+0.4–0.6%). Overall, mortality rate related to Marek’s disease was about 6% at the end of the cycle. Carcasses of chickens found during the occurrence of disease underwent necropsy, and tissues were collected to confirm the infection. Gizzard, cecal tonsil, intestine, spleen and tumor mass were collected and analyzed from a carcass of one hen, 32 weeks old and apparently asymptomatic. Multiplex real-time PCR performed on spleen tissues detected the presence of MD virus pathogenic strain. Macroscopic and microscopic evaluation of the rest of the samples confirmed the neoplastic disease. Moreover, the immunophenotype of the tumor cells was identified as CD3 positive by immunohistochemical (IHC) staining. The vaccinated flock had become rapidly infected with the MD virus, which proves that the challenge of the MD virus was too strong in the rearing house at the beginning of the cycle, causing the outbreak.

## 1. Introduction

Marek’s disease (MD) is one of the most ubiquitous diseases in poultry; it is a highly contagious and rapidly progressive lymphoproliferative disease that appears in a visceral and neurological form [1]. Commonly, MD is one of the most ubiquitous avian infections in chicken flocks worldwide. Every flock is presumed to be infected, except for those maintained under pathogen-free conditions. Although clinical signs are not always visible, in infected flocks a decrease in growth rate and egg production can be recorded during the infection, thus leading to severe economic losses. The main pathological changes during the infection are the neoplastic transformation of CD4+ T cells, which proliferate to form tumors, and immunodepression [2]. This disease has a massive economic impact on the poultry industry, despite great availability of commercial vaccines utilized to control it. Moreover, there are many concerns about MD vaccines because, although effective, they are live and imperfect vaccines, which do not always prevent infection with replication and shedding of pathogenic MD virus [3,4]. The causative agent of MD is an alphaherpesvirus, the gallid herpesvirus 2 (GaHV-2), a member of the genus *Mardivirus* of the Alphaherpesviridae subfamily [4], whose virulence is associated with its ability to induce lymphoproliferative lesions.

MD virus infects via the respiratory route, initially infecting lymphocytes and macrophages in the lung, then it is transported to the main lymphoid organs, the bursa of Fabricius, thymus and spleen, where it causes an acute cytolytic infection [5]. After its replication in B lymphocytes, this virus infects the activated T lymphocytes, mainly CD4+ cells. Molecular and cellular changes in these infected CD4+ T lymphocytes initiate the formation of lymphomas. During the infection, the pathogen is transported in the blood lymphocytes toward the skin, where it replicates mostly in feather follicles, from which it is spread into the environment [6]. Latent infection is characterized by the presence of the viral genome in cells accompanied with low antigen expression. MD tumor-associated surface antigen is present in lymphomas caused by MD, and it can be used for differential diagnosis [7]. There are three existent serotypes of *Mardivirus*: serotype 1 (chickens and turkey isolates) includes all virulent strains and some attenuated strains such as CVI988; serotype 2 (chicken isolates) is avirulent, not causing clinical disease and serotype 3 is commonly isolated from turkeys and avirulent [8].

Currently, there are available commercial vaccines to protect chickens from MD virus, and, due to the risk of early exposure of chicks to MD virus infection, they must be protected as soon as possible; therefore, vaccination in hatcheries is practiced worldwide [9,10]. Vaccinal protection is influenced by many factors including host genotype and individual susceptibility [11]. Current vaccines do not induce sterilizing immunity despite protecting chickens from developing tumors. Thus, MD field viruses may still cause infection in vaccinated chickens, and their replication allows shedding of fully infectious virions through skin dander and poultry dust. Indeed, it could be possible that the wide use of vaccines could drive evolution of the field strains toward greater virulence [12,13], which may be the situation in the present case. Generally, in attenuated vaccines, the attenuation procedure, dose per head [14] and route of administration are important determinants of vaccination success [15]. It is always recommendable to verify inoculation of the correct vaccine dose by utilizing serological and molecular analysis [16].

Identification of specific proliferative lesions is a highly suggestive sign in diagnosis of MD; lymphomatous lesions consist of development of foci characterized by small- and medium-sized lymphocytes and blast cells, always pleomorphic and various in size [17]. In MD virus infection, target cells of the malignant transformation are mainly CD4+ CD8- phenotype; therefore, immunohistochemical specific staining can help in differentiating the disease from lymphoid leucosis and other neoplastic pathologies [18].

This study reports an outbreak of MD occurring in a flock southeast of Sicily. In a large shed 22,000 Ross 308 chickens were raised from the beginning of March 2019 when, after 32 weeks, a weekly increase in mortality rate of 0.4–0.6% was observed. All chickens belonged to the same group of production and received in hatchery repeated vaccination against Marek’s disease (Rispens CVI 988 strain + HVT): in ovo three days before hatching and intramuscularly after hatching. Once hypothesized that the increase in mortality could be linked to MD virus infection, every single carcass found in the shed was analyzed to confirm the causes of death. Approximatively 2000 carcasses were analyzed by necropsy through the raising cycle. Postmortem examination showed gross lesions highly suggestive of MD virus infection in at least 1400 chickens. Samples of spleen from 20 carcasses were collected and subjected to real-time PCR in order to detect MD virus strain, according to the procedures reported below. Moreover, representative portions of the affected organs were collected to perform histological and immunohistochemical investigation. In addition, on a carcass of one female chicken, apparently asymptomatic, a neoplastic mass developed in the area below the cloaca was observed. The clinical suspicion of MD virus infection drove laboratory investigations for molecular detection of the MD virus strain and immunohistochemical characterization of suspected tumor. Laboratory results confirmed the cause of the lymphoproliferative lesion as attributable to MD infection. After ascertaining the nature of infection, the raising cycle was constantly monitored until the end, and all the carcasses found were submitted to necropsy to confirm the disease and discriminate the stage of infection. The presence of MD virus in the shed was observed until 47 weeks of age, and it matched an increase in weekly mortality rate up to 0.4–0.6%. MD virus infection resulted in a total mortality rate of 6% at the end of the cycle.

## 2. Case Report

In April of 2019, in the shed, a female chicken Ross 308, 32 weeks old and apparently asymptomatic, was analyzed after death. The hen showed a tumor under the cloaca of about 3 cm and a neoplastic proliferation in large tracts of intestine. To ascertain the nature of infection, representative portions of tumor mass, gizzard, intestine and cecal tonsils were collected and fixed in 10% buffered formalin until the histological processing. In addition, a portion of spleen and tumor mass were immediately frozen at −20 °C and sent to the laboratory for molecular screening (real-time multiplex PCR, Hangzhou, China) for suspected viral infection. Characterization of the tumor was performed to understand if it was related to viral infection.

Total RNA and DNA was extracted directly from tissues using the commercial spin columns kit “Kylt^®^ RNA/DNA purification” (AniCon Labor GmbH, Holtingausen, Germany) according to the manufacturer’s instruction. Two kits were utilized in order to detect the sequences of vaccinal strains and field strains: “Kylt^®^ MDV & Rispens DIVA” (AniCon Labor GmbH, Holtingausen, Germany) that allow to distinguish vaccinal strain (Rispens CVI 988) and wild MD virus strain; and “Kylt^®^ HVT” (AniCon Labor GmbH, Holtingausen, Germany) to detect turkey herpesvirus (HVT). The real-time PCR was performed according to the manufacturer’s instruction. Each reaction consisted of 4 µL of total DNA and 16 µL of reaction mix. The multiplex real-time PCR was performed on a LineGene 996 (Bioer, Hangzhou, China) according to the following conditions: 1 cycle at 95 °C for 10 min and 42 cycles at 95 °C for 15 s and 60 °C for 1 min.

In order to perform histological examination, 4 μm thick sections were obtained by formalin-fixed paraffin-embedded tissue that were set on slides treated with silane (3-aminopropyl-trieossi-silane) in order to avoid section detachment during staining. The preparations obtained were dried overnight in an oven at 37 °C, followed by dewaxing by xylene for 20 min. After a descending alcohol series (100%, 95%, 75% and 50%), slides were washed in distilled water and then stained with hematoxylin and eosin (HE). This was followed by the ascending scale of alcohols (50%, 75%, 95% and 100%) and clarification in xylene. After this phase, the slides were mounted in acrylic mounting medium (Eukitt^®^, O. Kindler GmbH, Freiburg, Germany). Morphology analysis was performed through a Leica DMLB microscope connected with a Nikon camera.

Additionally, in order to identify T or B cell markers of the tumor, immunohistochemical studies were performed on formalin-fixed paraffin-embedded tumor tissues, cecal tonsil and gizzard. Serial sections (4 μm thick) on glass slides were washed in xylene and hydrated in different (decreasing) concentrations of alcohol. After dewaxing, the slides were heated in a solution of sodium citrate (pH 6.0) at 96° C for 20 min for antigen retrieval. Endogenous peroxidase activity was blocked with 3% hydrogen peroxide for 30 min. Slides were treated with 1% bovine serum albumin (BSA) for 30 min and incubated for 1 h at room temperature in the presence of 0.1% BSA with an antibody against CD3 (Polyclonal Rabbit, 1:50, A0452 Dako-Agilent, Santa Clara, CA, USA) and an antibody against CD20 (Polyclonal Rabbit, 1:800, PA5-16701, ThermoFisher Scientific, Waltham, MA, USA) that identify T-lymphocytes (cytoplasmic region of the CD3ε-chain) and B-lymphocytes (C-terminus of CD20 protein). These antibodies were tested by several authors [19,20,21,22,23] that proved cross-reactivity with chicken T- and B-lymphocytes. After two rinses with phosphate-buffered saline (PBS), tissue sections were treated for 30 min with universal polymer labeled with horseradish peroxidase (MACH-1, Bio Optica, Milan, Italy), followed by the chromogen 3-3′ diamminobenzidine tetrahydrochloride (DAB) for 3 min and counterstained with Mayer’s hematoxylin. The specific primary antibody was replaced by PBS in tissue sections used as negative controls. The DAB reaction developed a brown precipitate when positive. All immunostained sections were analyzed using a Leica DMLB microscope equipped with a Nikon DS-Fi1 digital camera.

## 3. Results

This case report describes the occurrence of Marek’s disease virus infection in a flock southeast of Sicily. The flock consisting of 22,000 breeder chickens (20,000 females and 2000 males) showed symptoms at the beginning of the 32nd week of age, when the weekly mortality rate was around 0.5%. Similar values of 0.5–0.6% mortality were registered weekly until the end of the 47th week, when total mortality rate related to MD reached 6%. Mortality not attributable to MD was 6%, resulting in a total of 12% at the end of cycle.

This case was also characterized by an absence of evident clinical external signs, since chickens were apparently healthy and in good body condition. In fact, the suspicion of MD virus infection was made only after the evaluation of gross macroscopic and microscopic lesions at necropsy.

**Figure 1:**
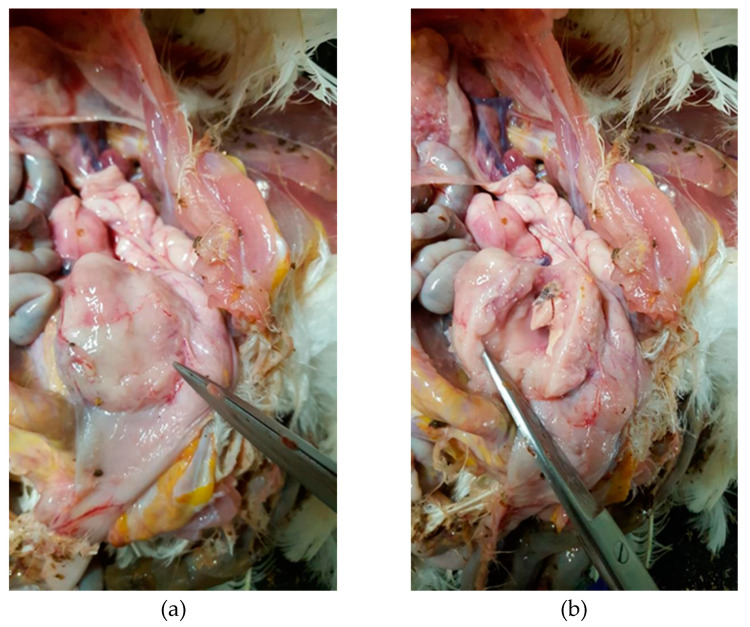
Tumor mass under the cloaca (**a**); internal part of tumor (**b**).

The hen necropsied showed gross lesions on visceral organs, mainly on the gastroenteric system, and the presence of a 3 cm length tumor, light-red colored outside and grey colored inside, with a fatty consistency, well protected under the cloaca region, that caused the overturning of the intestine, as shown in Figure 1. Initially, the external appearance of the carcasses did not give any indication of the severity of the clinical situation. The hen was clean, in a mediocre state of nutrition with crest and wattles normally developed. It did not present foot pad lesion, but at necropsy, the liver was slightly increased in volume with rounded edges, and the spleen was hyperplastic with evident trabeculation, triple in volume compared with hens of same age. There was no residue of bursa of Fabricius, the kidneys were slightly swollen with evident texture and the pancreas was increased in volume and pink colored. There was no other alteration on the rest of organs. After a careful analysis, the lesions could be addressed to a MD virus infection.

Generally, the other chickens showed similar gross lesions characterized by diffuse enlargement of the liver, kidney, spleen and ovary. Specifically, males showed hypotrophy of the gonads, and females showed atrophy of the ovary at times with bleeding follicles. In all the carcasses addressed to MD, the proventriculus was well defined and increased in volume with hypertrophy of the secretory glands with increased section thickness, the liver appeared with rounded edges, the kidney and spleen were dark and with fatty consistency and there was atrophy of the bursa of Fabricius and thymus.

Real-time PCR on spleen samples collected from 20 carcasses confirmed the suspicion of MD virus infection, detecting the presence of MD virus field strain, while all spleen samples were negative for both vaccine strains Rispens CVI 988 and HVT; therefore, the suspicion of MD viral infection was confirmed. The spleen and tumor mass from the hen of the case study gave the same results in real-time PCR, confirming that the tumor was undoubtedly attributable to MD virus infection, a natural virus-induced tumor model in chickens [1]. Considering the results of molecular screening associated with the macroscopic findings, in order to confirm if the tumor was really caused by the GaHV-2, it was necessary to thoroughly investigate the nature of the lymphoma according to the histological and immunohistochemical survey [24,25].

**Figure 2:**
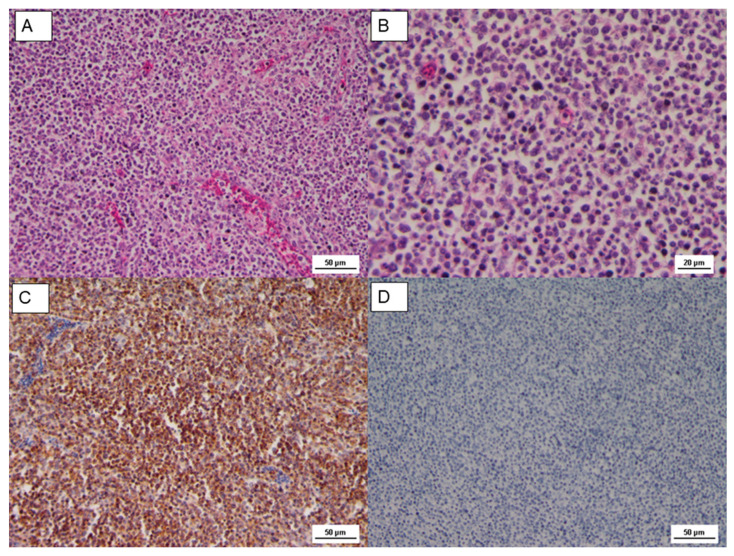
(**A**) Microscopic lesion of tumor mass, uniform proliferation of lymphoblast and small to medium lymphocyte, HE, Scale bar = 50 µm; (**B**) HE, Scale bar = 20 µm; (**C**) IHC CD3, diffuse positive staining, Scale bar = 50 µm; (**D**) IHC CD20, negative staining, Scale bar = 50 µm.

The histopathological findings on the tumor mass showed a uniform proliferation, consisting of lymphoblast, small to medium lymphocyte arranged in a diffused form. Tumor cells were characterized by large pleomorphic nuclei with prominent nucleoli (Figure 2). Lymphomas observed in our case were similar cytologically to A-type lesions [17]. The HE sections of proventriculus and gizzard showed moderate and multifocal infiltration in the mucosa and submucosa of lymphoid cells, mostly small in size and frequent mitotic activity; there was also a cellular infiltrate proliferation in the deepest glands. The proventricular mucosa, almost preserved, showed signs of catarrhal proventriculitis with bacterial aggregates. The same kind of infiltrate also affected the mucosal membrane of the muscular stomach, albeit with minor extension. These findings are compatible with the lymphoproliferative process [26].

**Figure 3:**
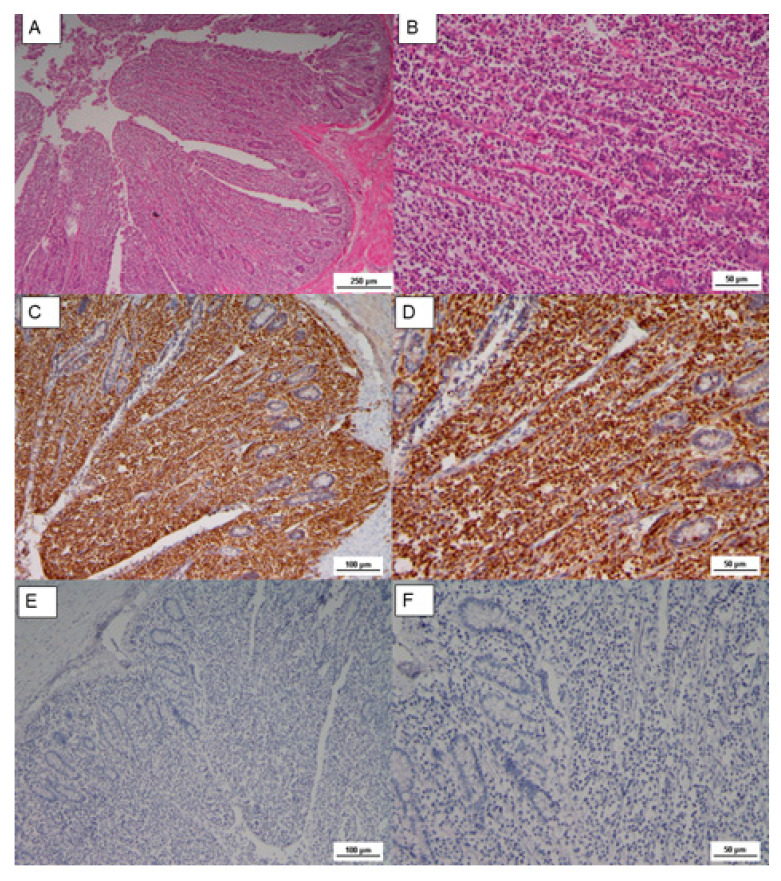
(**A**) Microscopic lesion of cecal tonsils, loss of intestine structure, caused by neoplastic proliferation and massive infiltration of lamina propria, HE, Scale bar = 250 µm; (**B**) Lymphoblasts with pleomorphic nuclei, and small to medium in size lymphocytes, HE Scale bar = 50 µm; (**C**) IHC CD3, diffuse positive staining, Scale bar = 100 µm; (**D**) IHC CD3 Scale bar= 50 µm; (**E**) IHC CD20, negative staining, Scale bar = 1000 µm; (**F**) IHC CD20, Scale bar = 50 µm.

A neoplastic and infiltrative proliferation was also observed in the cecal tonsils, where tumoral lymphoblastic cells replaced the intestinal mucosa, as shown in Figure 3. Lymphoblasts showed pleomorphic nuclei, but small and medium in size lymphocytes with dense chromatin were also observed.

The Cluster Differentiation 3 (CD3) is a protein complex and T cell co-receptor that is involved in activating both the cytotoxic T cell (CD8+ naïve T cell) and T helper cells (CD4+ naïve T cells). In the samples, most of these cells resulted in CD3 stained in a form of membrane precipitate, brown colored in the IHC analysis, as reported in Figure 2 and Figure 3. The immunophenotype of transformed cells was identified as CD3 positive by immunohistochemistry (Figure 2C and Figure 3C,D); in contrast, CD20 (B cell marker) was negative (Figure 2D and Figure 3E,F).

## 4. Discussion

Marek’s disease (MD) is an avian oncogenic lymphoproliferative infectious disease [27] causing huge economic loss in flocks worldwide because of its high mortality and morbidity. Although there are many different commercial vaccines to prevent MD neoplastic lesions and paralysis, there is subclinical circulation and shedding of MD virus in farms [28].

In this case report, the symptoms of MD occurred at the 32nd week of age with a weekly mortality of 0.5–0.6%, which was maintained until the 47th week. As reported by Atkins et al. [29], there is limited updated information on mortality rate related to MD generally in an unvaccinated flock of broiler breeders. It is estimated in the range of 0.6–23.4% between the 8th and 20th weeks [30]. In a vaccinated layer flock, average mortality rate is estimated around 5–8.2% between the 16th and 68th weeks [23], while in a vaccinated flock, in which during the trial animals showed several symptoms related to MD, it is estimated at 0.11–4.82% at the 40th week [31].

In this case report, mortality rate caused by MD virus infection reached 6%. Given that there is a discordance of values in literature, based on the status of infection and vaccination procedure, the present value nearly coincides with values reported by Zhuang et al. [32] in which the infection occurred in a vaccinated flock between the 24th and 30th week. The origin of infection could be attributable to an incorrect or incomplete vaccination procedure in the hatchery or to a massive viral challenge in the farm at the beginning of cycle. This hypothesis was confirmed by real-time PCR that identified a wild strain of GaHV-2 and ruled out the presence of vaccinal strains.

This case was also characterized by the absence of external signs with chickens apparently healthy. On the basis of this, the suspicion of MD virus infection was made only because of abnormal mortality rate registered and after the evidence of MD typical anatomo-pathological lesions at necropsy.

MD virus infection, even when not showing severe symptoms in chickens, infects an array of immune cells, especially T cells, CD4+ T cells being the most susceptible to the infection and the most likely to be transformed during disease course. This condition leads to the development of T cell lymphomas in peripheral nerves and visceral organs, which induces immunodepression [26]. However, presence of tumors is rarely observed in vaccinated flocks, meaning that only virulent strains can cause the onset of oncogenic modification. Furthermore, neoplastic masses are commonly observed in the liver, lung, spleen and heart [25].

In the female chicken of this case report, the main tumor mass was located under the cloaca; it showed, histologically, infiltrative lymphoblasts and small- to medium-sized lymphocytes. However, neoplastic transformations of lymphocytes were also widespread in other visceral organs, but no histological nerve lesions were observed, like some authors describe in certain strains of chickens [33]. The development of these neoplastic proliferations was attributable to a virulent strain of MD, as confirmed by real-time PCR. Morphological and immunohistochemical examination of tumor cells in Marek’s disease were described by Payne et al. [17], who identified three different types of lesions (A, B and C) in nerves affected. Lymphomas observed in our case were similar cytologically to the lymphoproliferations in the nerve A-type lesions, consisting in infiltrative lymphoblasts, large, medium, and small lymphocytes. It is also clear that the T cell population observed in this case has evolved toward malignant behavior [7,34,35]. Involvement of T cells in MD tumors was proved in the experiments of Sharma et al. [36], who induced T cell depletion in chickens and showed the development of tumors in birds after infection with MD.

Finally, as confirmed by data, pathologic changes observed in the visceral organs were referable to a typical MD infection, as described in the available textbooks [33].

## 5. Conclusions

Marek’s disease is widely controlled by vaccination programs, but sometimes chickens are not totally protected, mostly in the period immediately after the vaccination, when a strong challenge could interfere with the effectiveness of vaccination in absence of proper biosecurity practice. Moreover, the correct administration and dosage of the vaccine, especially when it is administered in ovo, is essential for the success of the vaccination, since sometimes the chicks are unable to develop immunity. In this case, thanks to molecular screening that confirmed the presence of a field strain and has ruled out the presence of vaccinal strains, it was clear that MD outbreak could be addressed to the erroneous procedures of vaccine dispensation or most probably to the presence of a virulent virus field strain circulating in the shed at the beginning of the raising cycle. In this report, the disease caused severe lesions and significantly increased the mortality rate, leading to an important economic loss. The importance of vaccination together with high biosecurity standards may minimize the economic losses related to a hidden infection such as Marek’s disease, only if followed by careful monitoring of vaccine efficacy. These findings provide the basis for the surveillance of MD virus, further study of its virulence mutants and the control strategies in Sicily.
